# Tear Osmolarity, Break-up Time and Schirmer’s Scores in Parkinson’s Disease

**DOI:** 10.4274/tjo.46547

**Published:** 2015-08-05

**Authors:** Esin Söğütlü Sarı, Rabia Koç, Alper Yazıcı, Gözde Şahin, Harun Çakmak, Tolga Kocatürk, Sıtkı Samet Ermiş

**Affiliations:** 1 Balıkesir University Faculty of Medicine, Department of Ophthalmology, Balıkesir, Turkey; 2 Balıkesir University Faculty of Medicine, Department of Neurology, Balıkesir, Turkey; 3 Adnan Menderes University Faculty of Medicine, Department of Ophthalmology, Aydın, Turkey

**Keywords:** Parkinson’s, tear, osmolarity, Schirmer’s, break-up time

## Abstract

**Objectives:**

Dry eye is an important problem in Parkinson’s disease (PD) with a potential to affect life quality. Tear osmolarity, accepted as the gold standard in dry eye diagnosis, has not been studied in this subset of patients so far. Therefore, in this study we aimed to evaluate tear osmolarity, Schirmer’s test scores and tear film break-up time (TBUT) in PD patients.

**Ma­te­ri­als and Met­hods:**

PD patients with a minimum follow-up of 1 year and healthy controls who admitted for refractive abnormalities were enrolled to the study. Subjects using any systemic medication with a possibility to affect tear tests were not included in the study. The presence of any ocular surface disorder, previous ocular surgery, previous dry eye diagnosis, any topical ophthalmic medication or contact lens use were other exclusion criteria. Age, gender, disease duration, and Hoehn and Yahr (H&Y) score for disease severity were noted, and blink rate (BR), Schirmer’s test score, TBUT and tear osmolarity of the right eye were measured in both groups.

**Re­sults:**

Thirty-seven PD patients and 37 controls were enrolled to the study. The groups were age and gender matched. The mean disease duration and H&Y score were 5.70±2.64 years and 1.70±0.93, respectively. H&Y staging and disease duration were not correlated to BR, Schirmer’s scores, TBUT, or tear osmolarity (p>0.05). The mean BR was 8.54±4.99 blinks/minute in PD patients and 11.97±6.36 blinks/minute in the control group. Mean Schirmer’s scores, TBUT and osmolarity values were 9.08±4.46 mm, 11.38±4.05 seconds and 306.43±12.63 mOsm/L in the PD group and 17.16±9.57 mm, 12.81±3.66 seconds and 303.81±16.13 mOsm/L in the control group. The differences were significant only in BR and Schirmer’s scores.

**Conclusion:**

BR and Schirmer’s scores decreased significantly in PD patients. Although not significant, the demonstrated tear osmolarity increment might be important to document the dry eye and inflammatory process of the ocular surface in PD patients.

## INTRODUCTION

Parkinson’s disease (PD) is a neurological disease specifically seen in elderly patients and is characterized by motor symptoms like bradykinesia, cog-wheel rigidity and resting tremor.^1,2^ The disease is also associated with non-motor abnormalities like autonomic and cognitive dysfunction, sleep and mood disorders, all seriously affecting the life quality of patients.^3^ Dry eye is a frequently encountered entity in PD due to reduced rate of blinking,^1,4^ which is an important and necessary process for the proper distribution of tears on the ocular surface and the prevention of tear evaporation. Seborrhea is also a common entity in PD which causes meibomian gland dysfunction and subsequently lipid layer abnormality in the tear film.^1^ The abnormal lipid layer disturbs the hydrophobic surface characteristics of the tear film, thereby increasing evaporation. Studies have also shown that meibomian gland dysfunction and evaporative dry eye are more prevalent in the elderly population.^5^ Therefore, age, reduced blinking rates, seborrhea and meibomian gland dysfunction all contribute to the increased frequency of evaporative dry eye in PD. Apart from evaporative problems of the tear film, studies have also demonstrated reduced tear secretions, probably due to autonomic dysregulation of the lacrimal gland.^6,7^

According to the International Dry Eye Workshop Study, dry eye is now considered to be inflammation of the ocular surface characterized by tear hyperosmolarity,^8^ which is now accepted as the gold standard of objective dry eye diagnosis and the single best marker of disease severity.^9,10^ In this study, we aimed to evaluate blink rate, Schirmer’s score, tear film break-up time (TBUT) and tear osmolarity in PD patients. To the best of our knowledge, this paper is the first to study tear osmolarity in PD.

## MATERIALS AND METHODS

The study was conducted under the approval of the institutional ethics committee and adhered to the tenets of the Declaration of Helsinki. All participants were informed about the study and informed consent forms were obtained. PD patients who met the United Kingdom Parkinson’s Society Brain Bank Clinical Diagnostic criteria11 with a minimum follow-up of 1 year with dopaminergic treatment (median 5 years, range 1-11 years) and age- and gender-matched controls admitted for refractive abnormalities with no other ocular or systemic pathology were enrolled to the study. PD patients using anticholinergic medication to control PD symptoms or for any other reasons were excluded from the study. Disease duration and severity, assessed with Hoehn and Yahr (H&Y)12 staging, was noted for PD patients. The H&Y stages from 1 to 5 are:

### Stage 1:

Only unilateral involvement, usually with minimal or no functional disability,

### Stage 2:

Bilateral or midline involvement without impairment of balance,

### Stage 3:

Bilateral disease: mild to moderate disability with impaired postural reflexes; physically independent,

### Stage 4:

Severely disabling disease; still able to walk or stand unassisted,

### Stage 5:

Confinement to bed or wheelchair unless aided.

Subjects using any systemic medication with a possibility to affect tear tests like steroids, hormonal drugs (androgens, hormone replacement therapy), beta-blockers, anticholinergic, antihistaminic, antipsychotic and antidepressive agents were not enrolled in the study. Participants with any ocular surface disorder, previous ocular surgery, previous dry eye diagnosis, or any topical ophthalmic medication or contact lens use were excluded. Demographic data including age, gender, ocular and systemic history were gathered; examinations involved only the right eyes of the patients. The examinations and measurements were performed between 9 and 11 a.m. in the order listed below.

### Blink Rate (BR)

A blink was defined as a bilateral paroxysmal closure of the eyelids (duration <1 second) in the absence of a provoking external stimulus. BR was defined as the average closure of the eyelids per minute in 5 minutes of video recorded while the patient was watching television. The average of two observers’ (A.Y. and E.S.) assessments was accepted as the final BR value.

### Tear Film Osmolarity

Tear osmolarity was measured using an in vitro diagnostic device (TearLabTM Osmolarity System, TearLabTM Corp., San Diego, CA, USA) designed to take a 50 nL sample of tears. We ensured that the system was functioning normally once per day with the electronic check cards as per the product manual instruction guide using monodose saline with an osmolarity value of 300 mOsm/L. The test was performed without anesthesia while the patient was looking straight ahead; the tip of the cartridge was touched to the tear meniscus and the reading was recorded.

### Tear Film Break-Up Time (TBUT)

After a 1 mg fluorescein-impregnated strip (Visimed, İzmir, Turkey) was moistened and placed into the lateral third of the lower eyelid, the interval between the last complete blink and the appearance of the first corneal dark spot in the stained tear film was measured 3 times and the average of the measurements was calculated.

### Schirmer’s Test

The Schirmer’s test (Madhu Instruments, New Delhi, India) was performed without anesthetic with the eye closed for 5 minutes after the strip was inserted into the lower conjunctival sac at the junction of the lateral and middle thirds, avoiding contact with the cornea, and the length of strip wetted in millimeters was recorded after 5 minutes.

### Statistical Analysis

Chi-square test was used to compare the gender differences of the groups. Student’s t-test was used to determine whether a statistically significant difference existed for age and tear film tests (TBUT, Schirmer’s test, osmolarity scores) between PD patients and controls. Level of significance was accepted as α=0.05. Pearson correlation was performed for correlation analysis of H&Y stage, tear film tests and BR.

## RESULTS

Thirty-seven PD patients and 37 controls were enrolled to the study. The mean age of PD and control patients was 67.41±7.03 (range, 44-77) years and 65.08±5.74 (range, 50-80) years, respectively. The male to female ratio in the two groups was 20:17 and 14:23, respectively, and there was no statistically significant difference between the groups with respect to age and gender (Student’s t-test and chi-square test, respectively, p>0.05). The mean disease duration and H&Y staging score were 5.70±2.64 (range, 1-11) years and 1.70±0.93 (range, 1-5), respectively. H&Y stage was 1 in 54.1%, 2 in 27.0%, 3 in 16.2% and 5 in 2.7% of the patients. The disease duration was significantly correlated with H&Y staging (Spearman correlation test, p=0.02 and r=0.390). However, H&Y staging and disease duration were not correlated to BR, Schirmer’s test, TBUT, or tear osmolarity (Pearson correlation test, p>0.05). In the PD group, BR was negatively correlated to TBUT (Pearson correlation test, p=0.04 r=-0.346). In comparison of the groups, significant differences were only seen in BR and Schirmer’s scores (p=0.01 and p<0.01, respectively). The mean BR, tear osmolarity, TBUT, and Schirmer’s test values are listed in Table 1. According to the results of our study, we performed post-hoc power analysis for BR and Schirmer’s scores, in which we found statistical significance. The analysis revealed that the power of the study was between 0.73 and 0.99 with the selected sample size of 37 patients in each group. 

## DISCUSSION

The most common ocular complaints of PD patients are related to ocular surface irritation, dry eyes and blepharitis.^13^ There are few studies related to dry eye and the ocular surface in PD patients and none of them evaluated tear osmolarity, which is now considered the gold standard and most reliable tool in dry eye diagnosis.^9,14^

There are different theoretical explanations for dry eye development in PD. One theory is that the autonomic dysfunction may affect the tear secretion and meibomian gland excretion of PD patients. The evidence that supports this hypothesis is the presence of Lewy bodies at sympathetic and parasympathetic ganglia as well as substantia nigra.15 The other most commonly accepted theory is that reduced BR causes tear film layer disruption, evaporation and dry eye.^1,4^

In the current study, the mean BR was 8.54±4.99 blinks/minute in PD patients and 11.97±6.36 blinks/minute in the control group; similar to previous studies, this difference was statistically significant.^4,15,16^ Decreased BR is closely related to dopamine activity in the central nervous system, and dopaminergic treatment can increase BR in PD.^17,18^ Studies have shown that the amplitude and velocity of blinks were also decreased in PD, indicating that the blinks are not only reduced in number but are also less effective.^19^

Effective blinking is necessary for appropriate excretion of lacrimal glands and for facilitating meibomian gland excretion. Meibomian glands secrete the outermost lipid layer of the tear film, which is critical for tear film stability and keeping the tear film on the ocular surface for longer periods. Therefore, seborrhea, insufficient meibomian discharge and disturbed tear discharge from lacrimal glands in PD might cause destabilization of the tear film, and the reduced blinking further facilitates the evaporation of this destabilized tear film. We also found a negative correlation with BR and TBUT, which supports the above-mentioned theories.

In our study, TBUT was lower in PD patients than the controls (11.38±4.05 vs 12.81±3.66 seconds) but the difference was not significant. Similarly, Reddy et al.4 evaluated PD and tear film tests and found no significant difference in terms of TBUT (8.4±1.9 vs 9.8±0.4 seconds). The TBUT of PD patients in Biousse et al.’s.^13^ study was lower compared to healthy controls. However, the latter study dealt with newly diagnosed and untreated patients and therefore the lack of dopaminergic treatment might have caused the difference in TBUT values between controls and PD patients.

We found a statistically significant difference in Schirmer’s scores of PD patients compared to the controls (9.08±4.46 vs 17.16±9.57 mm). This result is consistent with previous studies.^5,13,19^ As in the TBUT, untreated early-onset PD patients’ Schirmer’s scores were not statistically different from those of controls.^6^ The decrement in Schirmer’s scores might be due to ineffective and reduced blinking which causes decreased excretion from the lacrimal glands, or autonomic dysfunction with decreased innervational support to the secretory apparatus of the lacrimal functional unit. Another possible explanation is that decreased corneal sensitivity in PD causes lower BR, which was described by Reddy et al.^4^ The reduced corneal sensitivity, apart from its relation with blinking, might cause decreased neural impulses from the ocular surface to salivatory nucleus in the brain. Lower input to the salivatory center results in lower output from the secretory apparatus of the lacrimal functional unit. This might be another contributing mechanism in the reduced Schirmer scores of PD patients.

The reduction of quantity and quality of the tear film eventually resulted in increased osmolarity scores. We found a mean tear osmolarity of 306.43±12.63 mOsm/L in the PD group and 303.81±16.13 mOsm/L in the control group. Although the osmolarity was higher in the PD group, the difference was not statistically significant. In the setting of PD, both increased evaporation and decreased secretion have a role in the increment of tear osmolarity. Increased tear osmolarity triggers the inflammation-fibrosis-atrophy process in the ocular surface and the tear secretory glands of the lacrimal functional unit. Therefore, the osmolarity increment in PD patients reported in our study, although insignificant, is important as an indicator of ocular surface damage. To our knowledge, this is the first study to evaluate osmolarity in PD; as a result, we were unable to compare our osmolarity scores.

Correlation analysis of tear film tests, H&Y stage and disease duration did not reveal a significant association. Contrary to our findings, Tamer et al.1 found an inverse relationship between tear parameters and H&Y stage. Normally, a decline in tear film quality is expected as disease severity increases in PD patients. The reason we did not detect a correlation might be the unequal distribution of H&Y stage among the PD patients in our study (81.1% of the patients were either stage 1 or 2). If the distribution had been smooth or the sample size larger, the results might have been different. Therefore, larger sample sized studies are needed.

In conclusion, we demonstrated that BR and Schirmer scores decreased significantly in PD patients. Although not significant, the demonstrated tear osmolarity increment in this study might be a good indicator to document the dry eye and inflammatory process of the ocular surface in PD.

## Figures and Tables

**Table 1:**
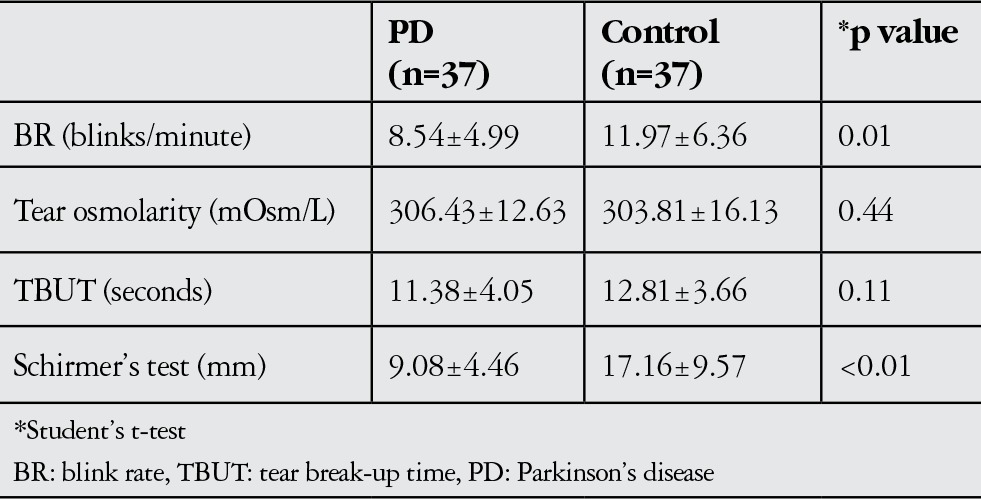
Comparison of blink rate, tear osmolarity, tear film break-up time, and Schirmer’s test scores between Parkinson’s disease and control groups

## References

[1] Tamer C, Melek IM, Duman T, Oksuz H (2005). Tear film tests in Parkinson’s disease patients. Ophthalmology.

[2] Duncan GW, Khoo TK, Yarnall AJ, O’Brien JT, Coleman SY, Brooks DJ, Barker RA, Burn DJ (2014). Health-related quality of life in early Parkinson’s disease: The impact of nonmotor symptoms. Mov Disord.

[3] Chaudhuri KR, Healy DG, Schapira AH (2006). Non-motor symptoms of Parkinson’s disease: diagnosis and management. Lancet Neurol.

[4] Reddy VC, Patel SV, Hodge DO, Leavitt JA (2013). Corneal sensitivity, blink rate, and corneal nerve density in progressive supranuclear palsy and Parkinson disease. Cornea.

[5] Guillon M, Maissa C (2010). Tear film evaporation-effect of age and gender. Cont Lens Anterior Eye.

[6] Kwon OY, Kim SH, Kim JH, Kim MH, Ko MK (1994). Schrimer test in Parkinson’s disease. J Korean Med Sci.

[7] Bagheri H, Berlan M, Senard JM, Rascol O, Montastruc JL (1994). Lacrimation in Parkinson’s disease. Clin Neuropharmacol.

[8] No author (2007). The definition and classification of dry eye disease: report of the Definition and Classification Subcommittee of the International Dry Eye WorkShop (2007). The ocular surface.

[9] Farris RL (1994). Tear osmolarity--a new gold standard?. Adv Exp Med Biol.

[10] Tomlinson A, Khanal S, Ramaesh K, Diaper C, McFadyen A (2006). Tear film osmolarity: determination of a referent for dry eye diagnosis. Invest Ophthalmol Vis Sci.

[11] Gelb DJ, Oliver E, Gilman S (1999). Diagnostic criteria for Parkinson disease. Archives of neurology.

[12] Hoehn MM, Yahr MD (1967). Parkinsonism: onset, progression and mortality. Neurology.

[13] Biousse V, Skibell BC, Watts RL, Loupe DN, Drews-Botsch C, Newman NJ (2004). Ophthalmologic features of Parkinson’s disease. Neurology.

[14] Miljanovic B, Dana R, Sullivan DA, Schaumberg DA (2007). Impact of dry eye syndrome on vision-related quality of life. Am J Ophthalmol.

[15] Goetz CG, Lutge W, Tanner CM (1986). Autonomic dysfunction in Parkinson’s disease. Neurology.

[16] Korosec M, Zidar I, Reits D, Evinger C, Vanderwerf F (2006). Eyelid movements during blinking in patients with Parkinson’s disease. Mov Disord.

[17] Agostino R, Berardelli A, Cruccu G, Stocchi F, Manfredi M (1987). Corneal and blink reflexes in Parkinson’s disease with “on-off” fluctuations. Mov Disord.

[18] Karson CN (1983). Spontaneous eye-blink rates and dopaminergic systems. Brain.

[19] Agostino R, Bologna M, Dinapoli L, Gregori B, Fabbrini G, Accornero N, Berardelli A (2008). Voluntary, spontaneous, and reflex blinking in Parkinson’s disease. Mov Disord.

